# First trimester myomectomy as an alternative to termination of pregnancy in a woman with a symptomatic uterine leiomyoma: a case report

**DOI:** 10.1186/1752-1947-5-571

**Published:** 2011-12-10

**Authors:** Kara Leach, Larissa Khatain, Kristina Tocce

**Affiliations:** 1 Department of Obstetrics and Gynecology, University of Colorado Denver School of Medicine, Mailstop 198-2, 12631 East 17th Avenue, Room 4209, Aurora, CO 80045, USA

## Abstract

**Introduction:**

Performing a myomectomy during pregnancy is extremely rare due to the risk of pregnancy loss, hemorrhage and hysterectomy. Favorable outcomes have been demonstrated with select second trimester gravid myomectomies. Literature documenting first trimester surgical management of myomas during pregnancy is scant. Patients with symptomatic myomas failing conservative management in the first trimester may be counseled to abort the pregnancy and then undergo myomectomy. Reports focusing on myomectomy in the first trimester are needed to permit more thorough options counseling for patients failing conservative management in the first trimester.

**Case presentation:**

A 30-year-old Caucasian primagravid (G1P0) was referred for termination of her pregnancy at 10 weeks due to a 14 cm myoma causing severe pain, constipation and urine retention. Her referring physician planned an interval myomectomy following the abortion. Instead, our patient underwent myomectomy at 11 weeks. Two leiomyomas were successfully removed; she delivered a healthy infant at term.

**Conclusion:**

Patients in the first trimester should not be counseled that termination followed by myomectomy is the best option for symptomatic myomas, failing conservative treatment. Management should be individualized after taking into account the patient's symptoms, gestational age and the location of the myomas in relation to the placenta. Any field providing women's health services will be impacted by the ability to offer more thorough options counseling for women with refractory myomas in the first trimester.

## Introduction

The prevalence of uterine myomas during pregnancy is estimated to be 0.3% to 2.6%, of which 10% result in pregnancy complications [1]. Complications include pregnancy loss, pelvic pain, placental abruption, hydronephrosis, premature rupture of membranes, preterm labor, intrauterine growth restriction, fetal malpresentation and postpartum hemorrhage. The prevalence of these complications is increased if there are multiple masses, if a myoma is retroplacental and if a myoma is larger than 3.6 cm in diameter (200 cm^3^) [1]. Conservative management is the first line of treatment during pregnancy and consists of bed rest, hydration and analgesics. If these measures fail, patients may be presented the option of induced abortion with myomectomy at a later date.

The literature consists mainly of case reports and retrospective studies [2-4]; there are few prospective studies of pregnancy-preserving myomectomies [5,6]. The majority of these surgeries were performed in the second trimester for intractable pelvic pain and had excellent outcomes: very few pregnancy losses and no hysterectomies have been reported. Traditional recommendations, including operating only on pedunculated myomas [2], only during the fourth and sixth month of pregnancy [7] or only during the 14^th ^to 15^th ^week [8], warrant re-evaluation.

Literature focusing on outcomes of myomectomies performed during the first trimester is scant [5,4,9]. This report of a first trimester myomectomy suggests that certain cases may also have the safety and advantages of second trimester myomectomy.

## Case presentation

A 30-year-old Caucasian primagravid (G1P0) was referred to our university hospital at 10 weeks and four days, for termination of the pregnancy due to a 14 cm myoma causing severe pain, constipation and urine retention. Six weeks following the abortion, she was to have a myomectomy with her primary physician.

During her first prenatal visit at eight weeks gestation, her uterus was noted to be larger than expected by dates. She was experiencing significant pelvic pain and constipation. An ultrasound showed a right-sided posterior 11.5 × 11 cm uterine myoma, displacing her uterus to the left. Two weeks later, a repeat ultrasound showed enlargement of the myoma to 14 cm in diameter; 2 cm of myometrium was noted to be between the myoma and the interuterine cavity. Our patient continued to have worsening pelvic pain, which was refractory to oral narcotics and nonsteroidal analgesics. She also experienced severe constipation; her last normal bowel movement was three weeks prior to presentation. An aggressive bowel regimen, including dietary modification, bisacodyl, magnesium citrate and enemas, was only minimally successful, and our patient remained considerably uncomfortable. Voiding every 60 minutes was also necessary to prevent urine retention. After two and a half weeks of outpatient medical management, she refused inpatient admission, indicating that she could not tolerate continued expectant management.

**Figure 1:**
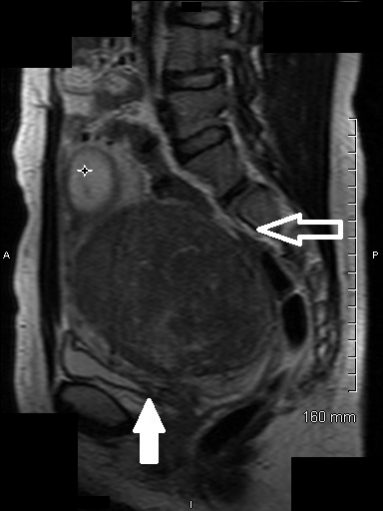
**Preoperative magnetic resonance imaging**. Magnetic resonance image of her pelvis without contrast shows a gravid uterus with pregnancy in the fundus (star), compression of the colon (empty arrow) and compression of the urethra (filled arrow) that were causing our patient's symptoms.

On presentation to our university hospital, magnetic resonance imaging was performed and showed compression of her colon, bladder and proximal urethra (Figure 1). The large fibroid was noted to be posterior and the placenta, anterior. During options counseling at our institution, our patient decided that she was unable to proceed with an induced abortion and opted to undergo a gravid myomectomy. She understood that elective surgery is typically postponed until the second trimester to minimize the fetal exposure to anesthesia and to reduce potential for fetal loss. Due to her tremendous discomfort, she decided to proceed with a first trimester myomectomy, accepting the risks of pregnancy loss, fetal injury and hysterectomy.

A myomectomy was performed at 11 weeks gestation via a vertical skin incision. Upon entry into her pelvis, her uterus was found to be displaced into the left lower quadrant, and the subserosal myoma obliterated the posterior cul-de-sac on the right. Vasopressin was injected into the capsule of the myoma (13 cm × 6 cm × 9 cm) to decrease operative blood loss, and the tumor was shelled out with a blunt dissection and Bovie cautery. Following removal of the dominant myoma, a second, smaller myoma (7 cm × 5 cm × 2.5 cm) with areas of cystic degeneration was discovered at the level of the internal cervical os. This was also resected. The uterine incisions were closed with multiple layers of 0 Polysorb. Excellent hemostasis resulted. The remaining pelvic and abdominal organs were inspected and found to be normal. The estimated blood loss was 500 cm^3^. Fetal heart tones were confirmed by ultrasound at the conclusion of the procedure; a moderate amount of post-procedure vaginal bleeding was noted. Our patient remained in the hospital for four days postoperatively and was discharged home having normal bowel movements, voiding regularly and with a reassuring fetal status. Although vaginal bleeding persisted throughout her postoperative course, her hematocrit remained stable and fetal heart tones were documented daily. Since our patient did not have symptoms of cramping, but vaginal bleeding was persistent, nonsteroidal anti-inflammatory drugs were not started. There was no disruption of the corpus luteum during the surgery, so progesterone supplementation was not administered. Our patient required routine narcotic postoperative pain management; intravenous patient-controlled analgesia was discontinued on postoperative day two. She was no longer requiring pain medication at her two week postoperative clinic visit.

Our patient returned to her primary physician, received routine prenatal care and the pregnancy progressed without complications. She was offered a vaginal trial of labor at our institution since the uterine cavity was not entered during the myomectomy; however, a primary Cesarean delivery was performed after she failed to go into spontaneous labor by 40 weeks and three days. She delivered a healthy girl with an Apgar score of nine and nine and a weight of 4356 g. Her postpartum course was uncomplicated.

## Discussion

Myomectomy during pregnancy has traditionally been reserved for cases of severe, intractable pain that have failed to be addressed by conservative management after the first trimester. Due to the potential risks during gravid myomectomy (including hemorrhage necessitating hysterectomy, pregnancy injury and/or pregnancy loss), patients in the first trimester with symptomatic myomas who fail to respond to conservative management may be offered induced abortion followed by interval myomectomy. For patients unwilling to undergo termination of pregnancy, gravid myomectomy is viewed as a last resort treatment.

Experience during the second trimester has suggested that myomectomy may be a safe and effective alternative if the myoma does not enter the uterine cavity. Multiple studies have shown that women who undergo surgical intervention in the second trimester actually have better outcomes than those who opt for conservative management [4-6] (Table 1). However, very few myomectomies have been reported during the first trimester, and it is uncertain whether the same safety and efficacy of the second trimester procedures can be extrapolated to earlier dates.

**Table 1:** Summary of studies, case reports and case series

First author	Type of study	Study details	Results/conclusions	Limitations
**Burton **[2]	Retrospective	n = 106 gravid patients with myomas → 14 ex-laps: six gravid myomectomies, all pedunculated with stalks < 5 cm in diameter; patients operated on for abdominal mass and pain or failed conservative management	Six myomectomies: one lost to follow-up; five term deliveries. Entire cohort: 75% live births, 21% S/TAB, 4% lost to follow-up, 13% PTL, 13% surgery	Size of myomas and GA at time of myomectomy not reported; cannot compare tx versus conservative tx with data presented
**Carolis **[3]	Retrospective (first and second trimester)	n = 18 (6 weeks to 24 weeks): Same surgical criteria as Mollica [5]; myoma size ranging 2 cm to 40 cm	14 term C/S; one assisted delivery at 36 weeks; one term vaginal delivery; one miscarriage one day post-operatively with infection; one lost to follow-up	Small sample size of patients with myomectomy in first trimester; one of whom was lost to follow-up
**Celik **[4]	Case series (second trimester)	n = 5 myomectomies after failing conservative management with mean GA of 18 weeks and myoma size ranging 10 cm to 20 cm	Mean GA at time of delivery was 39 weeks	Small sample size
**Mollica **[5]	Prospective (first and second trimester)	n = 106 gravid patients with myomas, 10 weeks to 19 weeks: 18 myomectomies for recurrent pain, large (> 10 cm) or 'rapidly growing' myomas, or 'medium-large' myomas in lower uterine segment or affecting placental site	Myomectomy versus conservative: pregnancy loss: 0% versus 13.6%; PROM: 5.6% versus 22.7%; preterm labor: 5.6% versus 21.6%; post-C/S hyst: 0% versus 4.5%	GA not compared to outcomes
**Lolis **[6]	Prospective (second trimester)	n = 622 gravid patients with myomas: 16 with complications of pregnancy → 13 myomectomies for rapidly growing, failing conservative management, and distance from endometrial cavity > 5 mm versus three expectant management; myomas ranged in size from 105 g to 2274 g	Myomectomy versus conservative: pregnancy loss: 8.7% versus 33.3% → Myomectomy: one SAB after surgery at 15 weeks and one C/S at 29 weeks for placenta previa Conservative: PPROM at 22 weeks with PPH requiring hysterectomy	Small number of patients with pregnancy complications due to myomas
**Makar **[8]	Case report (second trimester)	n = 1: 14 week pregnant patient presented with progressive lower abdominal pain and an ex-lap showed a 12 cm pedunculated myoma in the pouch of Douglas	'Gravid myomectomy should only be performed during 14th to15th weeks'	Conclusions limited to14 weeks to 15 weeks
**Bonito **[9]	Case series (first and second trimester)	n = 5 myomectomies for symptomatic patients whose myomas were resistant to conservative management	three spontaneous deliveries and two Cesarean sections	Small sample size

C/S: cesarean section; ex-lap: exploratory laparatomy; GA: gestational age; hyst: hysterectomy; PPH; postpartum hemorrhage; PPROM: preterm premature rupture of membranes; PROM: premature rupture of membranes; PTL: preterm labor; S/TAB: spontaneous/therapeutic abortion.

Mollica *et al*. [5] conducted a prospective study of 106 pregnant women with uterine myomas who were admitted for recurrent pain, large or rapidly growing myomas, medium to large myomas in the lower uterine segment or myomas deforming the placental site. Of these, the earliest gestational age to have undergone myomectomy was 10 weeks, but the number of first trimester procedures was not delineated in the report. Unfortunately, in this study, data is not available to compare specific gestational age to outcome. Regardless of gestational age, the outcomes for all women who underwent myomectomy (n = 18) was superior to those managed conservatively in terms of pregnancy loss (0% versus 13.6%), premature rupture of membranes (5.6% versus 22.7%), preterm labor (5.6% versus 21.6%) and post-Cesarean hysterectomy (0% versus 4.5%). Furthermore, in this small sample the risks of spontaneous abortion, intrauterine growth restriction and post-Cesarean hysterectomy were lower among the gravid myomectomy group compared to pregnancies not complicated by myomas.

Carolis *et al*. [3] retrospectively described myomectomies performed on 18 women at gestational ages of six to twenty-four weeks, using the same surgical criteria as Mollica [5] (Table 1). Of these 18 women, four were in the first trimester (at six, seven, eight and twelve weeks); these myomas did not affect the placental site and ranged from 2 cm to 15 cm. Only one patient presented with pain, as ours did; the others had rapidly growing pelvic masses necessitating surgical intervention and diagnosis. The six-week patient was lost to follow up; the others had full term, uncomplicated Cesarean deliveries.

In our case, our patient had severe pain, constipation and symptoms of urinary retention. After failing with conservative management, she elected for a myomectomy in the first trimester. This decision was made after reviewing the scant literature available on both first and second trimester myomectomies. Despite postoperative vaginal bleeding, the pregnancy was successfully maintained and she delivered a healthy infant at term. This plan differed significantly from her initial counseling to terminate the pregnancy and undergo an interval myomectomy.

## Conclusion

Counseling patients with myomatous uteri who fail conservative management in the first trimester is challenging. Limited evidence illustrates safe and effective second trimester myomectomy in select cases; however, it is unknown whether this can be extrapolated to first trimester cases. Our case demonstrates a successful pregnancy outcome after myomectomy in the first trimester; however, larger studies focusing on first trimester gravid myomectomies are needed. Until this data is available, management of refractory symptomatic myomas during pregnancy should be individualized after careful review of the imaging studies and thorough patient counseling. Induced abortion followed by interval myomectomy may not be the only option.

## Consent

Written informed consent was obtained from the patient for publication of this case report and any accompanying images. A copy of the written consent is available for review by the Editor-in-Chief of this journal.

## Conflict of interest

The authors declare that they have no competing interests.

## Authors' contributions

KL was a major contributor in writing the manuscript. LK performed the literature review and contributed to writing the manuscript. KT also performed the literature review and was a major contributor in writing the manuscript. All authors read and approved the final manuscript.
